# Supplementary material for: Abortion information-seeking experiences among women who obtained abortions in Kinshasa, DRC: Results from a qualitative study
Source: PLOS Glob Public Health. 2024 Feb 21;4(2):e0002383. doi: 10.1371/journal.pgph.0002383 (PMC10880974; doi:10.1371/journal.pgph.0002383)
Supplement: Attachment — Submitted filename: DRC-QualPaper-2023-Response-2023-08-21-CLEAN.docx [file pgph.0002383.s001.docx]

**Manuscript PGPH-D-23-00900: Response to Reviewer Comments**

Dear *PLOS Global Public Health* Editorial Review Committee and Reviewers,

We would like to thank you for reviewing our manuscript and giving us the opportunity to revise and resubmit it for consideration in *PLOS Global Public Health*. We appreciate the reviewers' insightful and detailed comments and incorporated the recommended changes in this revised draft. We believe these changes strengthen our manuscript and its potential contribution to the field of global public health. We appreciate your continued review and look forward to your final decision.

Best,

Denise Ngondo and co-authors

**REVIEWER COMMENTS**

**Reviewer #1**

I would like to thank the editor and PLOS Global Public Health for the opportunity to review this manuscript. The authors examined how women obtained information about abortion options in Kinshasa, DRC, by analyzing qualitative data collected from interviews. Key themes they found were 1) selective and narrow information search from the restricted range of their social network; and 2) heterogeneous information and stigmatizing interaction during the information search process. This study holds great significance as it contributes to generating high-quality evidence on the barriers and facilitators of accessing safe abortion, which is particularly relevant in light of recent policy changes and public debates taking place worldwide. The manuscript demonstrates strength in its methods and is effectively written. I have included specific questions and suggestions for the authors to consider.

*We thank the reviewer for their detailed constructive feedback and respond to individual comments below.*

***Major comments***

1. Methods, page 6, lines 119-120: The authors need to provide references and details to support their definition of recent abortion as reported abortion within the seven years prior to the survey. Please clarify the evidence or rationale behind this definition.

*We appreciate this comment. We restricted the eligible sample to respondents who reported an abortion in the last 10 years in the quantitative survey although we ultimately ended up with a sample of abortions that all occurred within the last 7 years. Our description of these abortions as “recent” was subjective and not based on any definition of “recent”. We have replaced “recent” with “within the last 10 years” in descriptions of the sample.*

2. While the liberalization of abortion laws in 2018 is a significant event in the study setting, the study results lack adequate contextualization. As the recent abortion was defined as abortion that occurred between 2015 and 2021 (recent seven years), the authors may leverage the pre-post setting. It would be interesting to see the differences in information-seeking practices before and after the abortion liberalization in 2018. Media exposure surrounding the legal changes may have influenced women's awareness of abortion options and sources of information. This context needs to be manifested in their analysis to identify how women who seek information for abortion in DRC understand and interpret the context.

*We thank the reviewer for this suggestion. We agree that a comparison of abortion information-seeking experiences between the pre- and post-liberalization periods would be an interesting analysis and would provide important insights into how liberalization of abortion laws affects the diffusion of information. Unfortunately, we did not specifically probe about respondent’s exposure to legal debates and how it may have contributed to information about abortion services.*

*The small sample size and diversity of circumstances surrounding the abortion does not allow for a pre-post analysis. In addition, given delays in implementation, it is unlikely that the law change would have resulted in immediate changes to abortion knowledge, service availability, and care seeking practices, making it difficult to decipher the impact of the liberalization. We nevertheless added a comment about this point in the discussion section: “While the present study is unable to evaluate how the change in the legal status of abortion in 2018 may have impacted information seeking, we anticipate the fear of social sanctions remains a major concern, especially given 19% of women in Kinshasa were unaware of the legal conditions for abortion in 2021, three years after the legal change (11).”*

3. The authors identify two themes and then several subthemes under each, but they could have provided a more in-depth understanding of the relationships between the sub-themes. Under “Role of women in one’s community” (pages 14-16), the final paragraph (lines 330-337) does not seem to be about the role of other women in their social network.

In addition, too many points are under this single subtheme (Role of women in one’s community), which could have been separated out and organized. I would suggest separating items under “Role of women in one’s community” and reorganizing them. For example, how the women utilize trusted people in their community or informal network (e.g., sisters, friends, and some other mature people who they could rely on) may be one subtheme, and then another subtheme may be the challenges and adverse consequences of the information seeking from the social network.

In addition, seeking information from healthcare providers may be a different theme. It is under the “Activation of the social network,” but it does not seem to be a social network strategy. Instead, it could be seeking information from formal resources, which may or may not be helpful. The challenges and barriers these women faced seem to be another important layer that should be noted because even women who seek complete and accurate information from healthcare providers might not obtain it due to more structural limitations.

*Thank you for this suggestion related to the presentation of our results section. We agree with this reviewer and have revised the structure of the results accordingly, renaming the theme/some subthemes and separating seeking information from healthcare providers as its own subtheme instead of nesting it within the social network information seeking (pages. 11-18). We believe these revisions greatly improve the clarity and flow of this section.*

4. Although low-quality guidance and information from providers emerged from their analyses, the Discussion section lacks substantial discussion on this matter. It would be helpful to discuss the quality of care, abortion providers’ behaviors as a trusted source of information and guidance of safe abortion care, and the practical implications arising from these findings.

*We greatly appreciate this important comment. We have included a paragraph on quality of care and provider behavior as sources of information in the discussion (lines 765-825).*

5. The authors briefly mentioned media on page 19, lines 397-401, but did not elaborate further. Even in the first theme, there was no discussion on media. Was media (traditional form or social media) not an important source of information in this study setting? It would be helpful for readers to add some details about the frequencies of different information sources in the participant characteristics section.

*We appreciate the reviewer’s point about the lack of elaboration on the role of media in women’s information-seeking trajectories. Few participants in our study indicate a reliance on media sources for information about their abortion care and, thus, media was not highlighted as a central component of either theme. As indicated in the results under the “Heterogenous information on procedures” sub-theme within the “Quality of interactions” theme (previously the “Heterogenous and stigmatizing advice” theme), we note that television was described by at least one participant as a source of information. We added clarification that no participants mentioned use of the internet or other social media networks to understand their options for abortion care (lines 637-640): “Media was rarely mentioned as a source of abortion information (and the internet or social media were never mentioned), providing only sporadic and incomplete exposure to abortion methods, as one participant shared, "I had pretty poor knowledge about abortion, but on TV I had once heard about curettage and abortion pills."*

*Following qualitative research methods, we highlight participants’ voices and synthesize themes in the results, instead of reporting frequencies or other quantitative presentations of data generated from the study. In the short quantitative survey we used to obtain women’s sociodemographic characteristics, we did not ask women about the source of information for their abortion care, which is why this information is not presented in Table 1.*

***Minor comments***

1. Page 5, lines 108-109: There are unnecessary sentences. Please proofread the manuscript before submission.

*We thank the reviewer for this comment. These lines have been removed from the revised manuscript.*

2. Page 8. Line 168: It appears that Appendix Table 1 is not on the supplement materials list. Please provide the necessary information.

*We have removed reference to this Appendix Table from the text, as this was outdated.*

3. Page 9, Table 1: Education level would help readers better understand the study sample. In addition, I suggest classifying the abortion year based on the year of abortion liberalization in DRC.

*We thank the reviewer for this suggestion. We have added education to Table 1, per this reviewer’s guidance, however, we did not conduct a pre-post analysis, given the limitations discussed above (Reviewer #1, Major Comment #2).*

**Reviewer #2**

Thank you for the opportunity to review this manuscript. This is a timely paper which has the potential to shape policy on improving access to accurate abortion information and services in DRC.

*We thank the reviewer for their thorough feedback and appreciate their view that this is a much-needed report on abortion in this context. We have done our best to address specific feedback the reviewer provided below.*

***Abstract***
1. Line 28 _Authors may need to write out DRC in full before using abbreviation.
Introduction

*We appreciate this suggestion. We revised this line and first mention of DRC in the resubmitted manuscript.*

2. Lines 59-60 Is this referring to unsafe abortion practices? If yes, I would recommend authors use unsafe abortion practices for the sake of consistency. The author should kindly define what the recommended methods are, so that readers can appreciate the non-recommended methods.

*We thank the reviewer for this important comment. We have provided additional information regarding what is meant by non-recommended method and source, which is based on the WHO’s safe abortion guidelines. We now refer to these abortions as unsafe for simplicity (lines 64-66) and have added a footnote that clarifies what methods and sources are considered non-recommended: “ In the cited paper, non-recommended methods are anything other than procedural abortion or medication abortion and non-recommended sources are non-clinical sources for procedural abortions, however, medication abortion pills could be obtained from any source."*

3. Is there data on the prevalence of knowledge of the recent liberalized abortion law among women in DRC? The background might benefit from this data, and how that may be impacting on abortion seeking information.

*We thank the reviewer for the excellent question on women's level of knowledge of abortion laws in DRC. The abortion component of the PMA survey provided us with information on women's level of knowledge of the laws in Kinshasa, and we have added a couple of sentences regarding these estimates (lines 152-156): “As of 2021, fewer than one in five (19%) women in Kinshasa knew that the law allows abortion in certain circumstances (11); this proportion was even lower among adolescents, women with a low level of education, and those from the poorest households (11). This lack of awareness can limit perceived options for information care seeking if women fear legal sanctions for pursing an abortion.”*

***Methods***
The authors suggest that the study population was heterogeneous, but was saturation assessed in this study? Could the authors add a sentence or two on how saturation was tested and what was found?

*We appreciate this question about our sample and thematic saturation. We clarify that we considered the study sample heterogenous, given the wide range of participants’ ages, residence, marital status, parity, and timing, method, and source of their recent abortion. Diversity of women’s characteristics across these factors is what makes our study sample heterogenous.*

*As for thematic saturation, we added a sentence to the methods to clarify that our research team applied inductive thematic saturation techniques in which we focused on the emergence or non-emergence of new codes/themes, not the completeness of the existing codes/themes, as guided by qualitative research principles (lines 357-363): “Inductive thematic saturation techniques were used to achieve saturation of themes and sub-themes. Research team members focused on identifying newly emergent codes and themes, instead of the completeness of the existing codes and themes, at each research team meeting until no new codes or themes emerged (22).” Our large sample size (n=52) also reinforces our understanding that the themes identified reflect widely shared experiences of information-seeking among participants.*

***Results***
Lines 420-432 Could these also be termed as attempted deterrent from health providers?

*We thank the reviewer for this question. We agree that the last quote included in the identified text highlights attempts at deterrence from health provider. The first two quotes, however, illustrate how providers counselled women on multiple aspects of abortion procedures, including the “pros and cons” and details related to potential side effects. We highlight attempts at deterrence (third quote in the identified text) in in the manuscript on pg. 21, lines 673-687, “The risks appeared to be amplified as a mechanism for dissuading women or reflected the reality of a context characterized by suboptimal quality of care” and have provided additional context around the last quote to emphasize that the counselling appeared to encourage women to avoid the procedure, instead of providing them with person-centered, evidence-based care.*

***Discussion***
The role of social stigma on participants’ pathways for information-gathering is critical as highlighted by the authors in lines 449-450. However, the authors concluding remarks do not contain any recommendations on how social stigma could be addressed even if there was high awareness about abortion legality in DRC and availability of safe abortions services at the community level.

*We appreciate this suggestion. We added the following text to our Discussion section, where we indicate suggested recommendations to reduce the stigma associated with induced abortions in the community: “Such public awareness campaigns, or related community-level outreach, must also address the stigmatization of abortion by providing information about the frequency of this common reproductive health event and the fact that all types of people have abortions.”*

**Additional Editor Comments:**

1. In the abstract, spell out DRC before the use of the abbreviation

*We thank the editor for this suggestion. We revised this line and first mention of DRC in the resubmitted manuscript.*

2. In the introduction paragraphs

a. Any time restriction on abortion?

*We thank editor for this question. We now clarify that abortion is legally accessible through 14 weeks gestation in the first paragraph of the intro where we describe the abortion law.*

b. What is the primary access to abortion care in Kinshasa?

*We have added details regarding the most common source of abortion (and most common method) (lines 61-66): “There were an estimated 105 (95% CI 79-132) abortions per 1,000 women aged 15-49 in Kinshasa, the capital of DRC, in 2021, most often involving procedural abortion and private facilities (6). In addition, 20% of abortions in Kinshasa involved self-managed medication abortion (6), a safe alternative to facility-based care (7). However, approximately one-third of abortions remain unsafe, involving non-recommended methods and sources^[1]^ (6), increasing the risks of maternal morbidity and mortality.”*

c. How much does it cost? Is it affordable to most women, or is it privileged access?

*The editor raises a great point. The cost likely varies considerably, particularly in the private sector where most women report obtaining their abortions. However, we identified some information on the typical cost of safe abortion care in the DRC; a manual intra-uterine vacuum aspiration procedure costs between 11 and 17 USD, and misoprostol costs between 5 and 75 USD, depending on the supply method (*[*https://safe2choose.org/fr/abortion-information/countries/drc*](https://safe2choose.org/fr/abortion-information/countries/drc)*).*

*These costs are too high for many women, the DRC is one of the five poorest nations in the world. In 2022, around 62% of the country's population lived on less than $2.15 a day. Nearly one in six people living in extreme poverty in sub-Saharan Africa live in the DRC (*[*https://www.banquemondiale.org/fr/country/drc/overview*](https://www.banquemondiale.org/fr/country/drc/overview)*).*

d. The authors mentioned the stigmatisation around abortion. Are any cultural elements specific to the DRC population?

*We know that cultural elements, such as family shame and dishonor, and membership of religious groups totally opposed to giving birth before marriage, are not specific to the DRC but to several countries (Les grossesses non désirées et les avortements au Burkina Faso, en Palestine, au Pérou et en République démocratique du Congo: analyse comparée des déterminants socioculturels et communautaires, 2016, Médecins du monde). We were unable to identify research on DRC-specific factors contributing to the stigmatization of abortion in this context, however, we added the following sentence regarding religion that we suspect may be a factor: “Fear of stigmatization may be greater in a highly Catholic country, like the DRC, given the church’s prominent role in society and its opposition to abortion.”(***Lison** Guignard, « Résistances catholiques au protocole de Maputo. Mobilisations et controverses autour de la libéralisation de l’avortement en Afrique », Genre, sexualité & société [En ligne], 18 | Automne 2017,).

e. Does the decriminalisation of abortion reduce the number of unsafe abortions? What is the number of unsafe abortion care since the decriminalisation?

*We appreciate this pertinent comment. We know that restrictive laws don't prevent women from having abortions as abortion rates are similar across legal contexts, however, abortion legal restrictions are associated with more unsafe abortions. We added text to address this relationship in the first paragraph of the introduction: “While permissive abortion laws are generally associated with greater abortion safety (5), many abortions occurring in the DRC remain unsafe.”*

*There are no data to evaluate declines in abortion safety since decriminalization as there have been no national population-based studies of abortion in the DRC. However, we provide the rate of abortion and percent that were unsafe from the Akilimali et al 2023 study, which gets at this question for the province of Kinshasa, specifically.*

f. What prompts people to use unsafe options before and after decriminalisation? What are the barriers?

*This is an important question. We have no quantitative information on the specific obstacles that existed before the law was liberalized, but assume the absence of legal healthcare services is a primary factor. The PMA survey that included an abortion module was conducted after liberalization but indicates that only 19% of respondents knew that the law allows abortion in certain circumstances. We included this information in the background, which now reads: “As of 2021, fewer than one in five (19%) women in Kinshasa knew that the law allows abortion in certain circumstances (11); this proportion was even lower among adolescents, women with a low level of education, and those from the poorest households (11). This lack of awareness can limit perceived options for information care seeking if women fear legal sanctions for pursing an abortion.”*

g. What indicators does the legalisation of abortion bring public health good?

*We think the relationship between abortion law and abortion safety provide compelling evidence regarding abortion legalization being a public good. This sentence briefly addresses this relationship: “While permissive abortion laws are generally associated with greater abortion safety (5), many abortions occurring in the DRC remain unsafe.”*

h. What is the source of information before and after the decriminalisation?

*There is no empirical research specifically addressing this issue of how sources of information pre- and post- abortion decriminalization in the DRC changed to our knowledge.*

3. ***Methods***:

a. Remove the sentence, “I thought it was mentioned. It not, this is from.”.

*We have deleted those sentences that did not fit with the content of this paragraph in the revised manuscript.*

b. Have the survey results been published? Citation is missing.

*The results of the quantitative study have been published, which we now cite in this section. Here is the reference: Akilimali, P. et al. (2023) “Estimating induced abortion incidence and the use of non-recommended abortion methods and sources in two provinces of the Democratic Republic of the Congo (Kinshasa and Kongo Central) in 2021 : results from population-base , cross-sectional surveys of reproductive-aged women”, Sexual and Reproductive Health Matters, 31(1), p. 1‑23. We have added the article reference in the revised manuscript.*

c. How did the research team select to contact 65 women from the 93 women who reported having had an abortion between 2015 and 2021? What were the inclusion and exclusion criteria?

*Thank you for this question. We have clarified how we came to contact 65 women from the 93 eligible. The text now reads: “From the 93 eligible participants, interviewers first attempted to contact those who had the most recent abortions, continuing to contact women until reaching the goal of 50 completed interviews. In total, interviewers attempted to contact 65 women, among whom 52 women provided written informed consent to participate in an in-depth interview about their abortion experience and allow a digital recording of the interview.”*

d. Line 131- 132. The authors mention the four-day training about research ethics, informed consent etc. Of the nine interviewers, what are their prior backgrounds? Any research exposure or community background?

*We now clarify that all nine interviewers resided in Kinshasa and had prior data collection experience via the PMA project.*

e. Interviews were conducted in what language? French?

*Interviews were conducted in Lingala, widely spoken in Kinshasa. This is mentioned in the manuscript (Line 196).*

f. Ethical Concern – some women mentioned their use of abortion, even if it is not legalised, during the interview. How are women be protected from this research?

*We thank the editor for this question. In the methods section we added to our existing text regarding the ethical principles used in this research and how women were protected in this research: “Confidentiality and anonymity were guaranteed during each participant’s informed consent process, and participants were not identified by name or any other identifier in study materials. Data collected from audio recordings of in-depth interviews were immediately downloaded onto a password-protected computer, then erased from the recording device. The interviewers' notes were archived in secure cupboards in locked offices on the premises of the local DRC team. No personal information about respondents was included in the interviewers' notes.”*

4. ***Results and Discussion***:
a. Have any interviewees obtained more than one abortion in their lifetime? It is not mentioned in the table.

- *The women were not asked this question thus we cannot include it in Table 1.*

b. Among interviewees who have obtained abortion before and after the legalisation, have they had different lived experiences regarding obtaining information or care?

- *The editor raises and important research question however we only collected information about women’s most recent abortion experience and had no information about prior abortions.*

c. What are the typical family dynamics in obtaining abortion care? Open disclosure? Hiding?

- *Hiding best reflects typical family dynamics in seeking abortion care. We explain this by the fact that it is rare to find a good appreciation of clandestine abortion, even if we recognize its recurrence and high incidence. Overall, therefore an unfavorable perception of abortion, certainly due to religion and social morality. The attitudes of those who condemn abortion revolve around the following propositions: it's not right, it's not good, it's a crime, abortionists are murderers, abortion is murder. (Déterminants sociaux culturels et communautaires des grossesses non désirées et des avortements, Kinshasa, Médecins du monde, 2016).*

d. A rather large percentage of interviewees are never married and obtain an abortion. Any cultural or religious factors to this vulnerable population?

- *We don’t explore this in our study, but we see in the quantitative results from this study that abortion rates are much higher among unmarried compared to married women in this context, Abortion rates are also higher among women with greater education, thus the greater number of unmarried women obtaining abortions (and the corresponding greater number in our qualitative sample) is likely related to the conditions under which many women week abortion in order to remain in school.*

e. The authors provided many findings covering multiple themes, social networks and capitals to obtain information, stigma, and heterogeneous procedure information. Discussion, however, is somewhat thin without an in-depth extrapolation. Authors are suggested to expand the elaboration by 1-2 paragraphs.

*We contextualize key thematic findings regarding the selective nature of disclosure and how the fear of stigma informs this process. We have added a few paragraphs to contextualize the poor information received from the medical establishment and associated recommendations, which was not fully addressed in existing paragraphs (lines 717-834):*

*“Fear of social stigma and rejection shaped participants’ pathways for information-gathering, echoing findings from other contexts (15-17). These concerns reflect a reality for many women, who are often ostracized, isolated, and sometimes forced to leave their communities if they are found to have had an abortion (23-25). While the present study is unable to evaluate how the change in the legal status of abortion in 2018 may have impacted information seeking, we anticipate the fear of social sanctions remains a major concern, especially given 19% of women in Kinshasa were unaware of the legal conditions for abortion in 2021, three years after the legal change (11). To avoid these social sanctions, women were highly selective about to whom they disclosed their pregnancies, often describing the source of their trust and confidence in their selected informant(s). While several studies link fear of stigma to limited pregnancy disclosure (19, 26, 27), women’s decision-making in these scenarios prioritized privacy even in the absence of complete or accurate information about abortion options. These results suggest the need for public awareness campaigns and community-level outreach to inform people about the risks of unsafe abortion and the availability of legal reproductive health services to reduce these risks, which affect many families in the community.*

*Beyond awareness campaigns, efforts should address the stigmatization of sexual and reproductive health services, including abortion care, at the community level to improve women’s access to safe abortion care. One of the ways women seek information while avoiding public exposure is to involve their partners who don’t face the same risk of social shame. As suggested in other studies conducted in legally restricted abortion settings (12, 28), we found male partners take on much responsibility in women’s abortion trajectories, often connecting their pregnant partners directly to healthcare providers. However, in doing so, our results indicate some partners infringed on women’s reproductive autonomy, making decisions without sharing information.*

*Altogether, few women in our study knew about safe options to terminate their pregnancy, which corroborates the quantitative results showing only 23% of women were aware of a recommended method for abortion (11). Even in case of interactions with healthcare providers, our study revealed low-quality advice and information from healthcare providers. This may be explained by the lack of comprehensive, woman-centred abortion care and a lack of training in recommended abortion methods. Other research has shown low levels of medication abortion knowledge and inadequate information provided to women among pharmacy providers or drug sellers, which is consistent with our findings (29, 30). These results highlight the need to monitor the implementation of legal extensions to safe abortion care in the DRC, given concerns about low quality of care received. This is consistent with recent findings suggesting inadequate safe abortion care service availability and readiness to provide quality care in 2021 in Kinshasa (31). In addition, the provision of legal abortion care should consider task shifting opportunities, including the training of pharmacist who are among the most popular contact points for women seeking information on abortion care. The training of mid-level providers and pharmacists is in line with current WHO guidelines on safe abortion care and has the potential to significantly increase the quality of safe abortion care, thereby reducing abortion-related complications."*

1. In the cited paper, non-recommended methods are anything other than procedural abortion or medication abortion and non-recommended sources are non-clinical sources for procedural abortion, however, medication abortion pills could be obtained from any source.
